# Atoh7 promotes the differentiation of retinal stem cells derived from Müller cells into retinal ganglion cells by inhibiting Notch signaling

**DOI:** 10.1186/scrt305

**Published:** 2013-08-14

**Authors:** Wei-tao Song, Xue-yong Zhang, Xiao-bo Xia

**Affiliations:** 1 Department of Ophthalmology, Xiangya Hospital, Central South University, Changsha 410008, China

**Keywords:** Müller cells, Retinal ganglion cells, Atoh7, Notch, Stem cells, Differentiation

## Abstract

**Introduction:**

Retinal Müller cells exhibit the characteristics of retinal progenitor cells, and differentiate into ganglion cells under certain conditions. However, the number of ganglion cells differentiated from retinal Müller cells falls far short of therapeutic needs. This study aimed to develop a novel protocol to promote the differentiation of retinal Müller cells into ganglion cells and explore the underlying signaling mechanisms.

**Methods:**

Müller cells were isolated and purified from rat retina and induced to dedifferentiate into retinal stem cells. Next the stem cells were transfected with lentivirus PGC-FU-GFP or lentivirus PGC-FU-Atoh7-GFP. In addition, the stem cells were transfected with Brn-3b siRNA or Isl-1 siRNA or treated with Notch inhibitor gamma-secretase inhibitor (GSI).

**Results:**

The proportion of ganglion cells differentiated from Atoh7-tranfected stem cells was significantly higher than that of controls. Knockdown of Brn-3b or Isl-1 inhibited, while GSI promoted, the differentiation into retinal ganglion cells. Atoh7 promoted the expression of Brn-3b and Isl-1 but inhibited the expression of Notch1.

**Conclusions:**

Atoh7 promotes the differentiation of Müller cells-derived retinal stem cells into retinal ganglion cells by inhibiting Notch signaling, thus opening up a new avenue for gene therapy and optic nerve regeneration in glaucoma.

## Introduction

Glaucoma is a group of eye diseases characterized by the selective and progressive death of retinal ganglion cells, which in turn, causes a severe loss in visual function. A common treatment for glaucoma is the reduction of intraocular pressure (IOP), but this approach does not prevent the visual loss caused by the death of retinal ganglion cells (RGCs) [1,2]. In the late stage of glaucoma as many as 90% of retinal ganglion cells could be damaged. Numerous approaches have been developed to provide protection to ganglion cells, such as improving optic microcirculation, glutamate pathway inhibitors, neurotrophic factors, induction of heat shock protein expression and antioxidant therapy [3-5]. However, no effective treatments can essentially prevent ganglion cells and the optic nerve from being injured. Stem cells have been proposed as a new approach for the regeneration of ganglion cells [6,7]. However, retinal stem cells only exist in the pigmented ciliary epithelium and they are far too few to meet the clinical need [6]. On the other hand, the use of other stem cells, such as embryonic stem cells and neural stem cells, is greatly restricted due to ethical issues and graft rejection. Therefore, it is urgent to identify new types of retina-derived cells that are abundant in seed cells for the regeneration of ganglion cells.

Müller cells are the sole glia cells generated by retinal stem cells/progenitors [8]. Müller cells extend from the inner limiting membrane to the external membrane throughout the entire retina. The neurites of Müller cells are widely distributed in various types of neuronal cell bodies in the retina, and make extensive contacts with the retinal nerve neurons [9,10]. Extensive studies in recent years have implicated retinal Müller cells as potential retinal stem cells and various types of stimuli have been shown to induce the dedifferentiation of retinal Müller cells in zebrafish, chickens and adult rats [11-13]. These dedifferentiated retinal Müller cells exhibit the characteristics of stem cells, and can differentiate into neurons, including retinal ganglion cells. However, the proportions of ganglion cells induced in the above studies are too low to replace and compensate the dead and damaged cells in glaucoma. Therefore, strategies to enhance the differentiation of Müller cells into ganglion cells may hold promise for glaucoma treatment via optic nerve regeneration.

The induction and differentiation of retinal stem cells are largely regulated by the joint action of extracellular and intracellular factors. The family of transcription factors bHLH (basic helix-loop-helix) plays an important regulatory role in the differentiation of retinal cells. The vertebrate bHLH family includes the original nerve (proneural) gene, the Ath family, Ash family and Hes family for the antagonizing proneural gene [14]. Ath5 is one member of the Ath family, and Atoh7 is an expression form of Ath5 in the rat, which is involved in the differentiation of rat retinal ganglion cells. Atoh7 is an essential transcription factor that determines the competence state of retinal ganglion cell progenitors [15,16]. Ectopic expression of Atoh7 significantly increased the number of retinal ganglion cells differentiated from *in vitro* cultured retinal stem cells [17]. Therefore, we hypothesize that Atoh7 may also promote the differentiation of stem cells dedifferentiated from retinal Müller cells into ganglion cells.

The differentiation into retinal ganglion cells induced by Atoh7 is a complicated process, in which various kinds of genes and signal pathways interact. Recent studies have shown that Brn-3b, a downstream target gene of Atoh7, and Isl-1, a member of LIM-HD family, could synergize to promote the growth and differentiation of rat retinal ganglion cells during the embryo period [18]. On the other hand, the Notch signaling pathway negatively regulates the growth and differentiation of retinal ganglion cells. It has been shown that downstream effectors of Notch signaling regulate retinal ganglion cell differentiation [19].

In this study, we cultured rat retinal Müller cells *in vitro* and induced them to dedifferentiate into stem cells with a stem cell-conditioned medium. Next, we ectopically expressed Atoh7 in these cells to induce them to differentiate into ganglion cells. In addition, we interfered with the expression or activity of Brn-3b, Isl-1 and Notch1 in rat retinal stem cells, and examined the effects on their differentiation into ganglion cells, in order to explore the signaling mechanisms that regulate the re-differentiation of stem cells derived from Müller cells into ganglion cells.

## Material and methods

### Ethics statement

The use of animals in this study was in accordance with the Guidelines for Animal Experiments of Central South University, Changsha, China. All animal experiments in this study were conducted with the approval of the Animal Research Committee, Xiangya School of Medicine, Central South University, Changsha, China (Permit No. SCXK 2006–0002).

### Müller cell culture

The enrichment of the Müller cells was performed as previously described [11]. Briefly, the eyes from Day 21 Sprague Dawley (SD) rats were enucleated and washed several times with a phosphate-buffered solution (PBS) (Gibco: Grand Island, NY, USA). The retinae were dissected carefully to avoid contamination from the lens, the retinal pigment epithelium (RPE) and the ciliary epithelium. The retina was mechanically dissociated into small aggregates and trypsinized with 0.25% trypsin-EDTA (Sigma: St. Louis, MO, USA) in a 37°C incubator for 20 minutes. The digested retina was suspended in DMEM containing 20% FBS and 1:100 penicillin/streptomycin (Sigma), and inoculated in a 25 cm^2^ culture flask (Corning: Corning city, NY, USA) for five to seven days, until the Müller cells attached to the bottom of the flask. The cells were trypsinized and cultured in DMEM containing 20% FBS for six days to further purify the Müller cell population. Cells of the third passage were dissociated with 0.25% trypsin-EDTA and cultured in a serum-free dedifferentiation media containing DMEM/F12 (GIBCO), 1 × N2 supplement (GIBCO), 2 × B27 supplement (GIBCO), 20 ng/ml EGF (Peprotech: Rocky Hill, NJ, USA), 10 ng/ml bFGF (Peprotech), 2 mM L-glutamine (HyClone: Logan, UT, USA), 100 U/ml penicillin and 100 μg/ml streptomycin at a density of 1 × 10^5^ cells/cm^2^ for five to seven days to generate neurospheres. Half of the dedifferentiation media was changed every other day. The suspended and semi-suspended neurospheres were collected and dissociated with Accutase (Sigma), and then cultured in serum-free dedifferentiation media to obtain a purified generation.

### Immunohistochemical analysis

Immunocytochemical analysis was performed as previously described [20]. Briefly, 4% paraformaldehyde-fixed cells were incubated in PBS containing 3% bovine serum albumin (BSA), 5% goat serum and 0.3% TritonX-100 at 37°C for 1 h, followed by incubation at 4°C overnight with the primary antibodies listed in Table 1. The cells were then incubated in the dark at room temperature for 1 h with secondary antibodies anti-rabbit IgG conjugated with FITC (Sigma), anti-mouse IgG conjugated with FITC (Sigma), anti-rabbit IgG conjugated with TRITC (Sigma) and anti-mouse IgG conjugated with TRITC (Sigma). Finally, the cells were incubated with DAPI (Beyotime: Shanghai. China) for five minutes and images were captured using fluorescent microscopy (Leica: Solms, Germany).

**Table 1:** List of antibodies used in this study

**Antibodies**	**Species**	**Dilution**	**Cell markers**	**Company**
**GS**	Rabbit	1:200	Müller cells	Sigma
**Pax6**	Rabbit	1:100	Retina stem cells	Santa Cruz
**Nestin**	Mouse	1:100	Retina stem cells	Abcam
**Notch1**	Mouse	1:200	Retina stem cells	Sigma
**Sox2**	Rabbit	1:100	Retina stem cells	Sigma
**Thy1.1**	Rabbit	1:200	RGCs	Sigma
**Brn-3b**	Rabbit	1:300	RGCs	Santa Cruz
**PKC**	Rabbit	1:200	Bipolar cells	Sigma
**Rhodopsin**	Mouse	1:200	Photoreceptor cells	Abcam
**HPC-1**	Rabbit	1:200	Amacrine cells	Sigma
**calbindin**	Rabbit	1:100	Horizontal cells	Sigma
**Edu**	Rat	1:1,000	Retina stem cells	RiboBio

### FACS analysis

The purity of the enriched Müller cells was examined by fluorescence-activated cell sorting (FACS) analysis using antibodies specific to Müller cells. Briefly, the cells in the monolayer culture were dissociated into single cells by the method of passage and centrifugation. The cells were fixed with 4% paraformaldehyde and blocked with a PBS containing 1% BSA and 0.1% TritonX-100 for 30 minutes at 4°C, then incubated at 4°C for 1 h with the primary antibodies listed in Table 1. After that, the cells were incubated in a PBS-BSA solution containing the appropriate secondary antibodies linked to FITC at 4°C for 1 h in the dark. The cells were washed with PBS and re-suspended in PBS for FACS analysis.

### Edu labeling analysis

To evaluate the proliferation of stem cells, neurospheres were incubated with 1:1,000 Edu (RiboBio: Guangzhou, Guangdong, China) diluted in culture solution overnight at 37°C. After several washes, the cells were fixed with 4% paraformaldehyde for 30 minutes. Fixed cells were incubated with an Apollo buffer (RiboBio) for 30 minutes at room temperature in the dark. The cells were washed with 0.5% TrixtonX-100 (diluted in PBS) for 10 minutes, followed by staining with Hoechst 33342 (RiboBio: Guangzhou, Guangdong, China) at room temperature for 30 minutes in the dark. Images were captured using fluorescent microscopy.

### Lentivirus PGC-FU-Atoh7-GFP constructs and transfection

Atoh7 expression vector Lentivirus PGC-FU-Atoh7-IRES-GFP was constructed by GENECHEM (Shanghai, China). The neurosphere cells were transfected with the lentivirus at the multiplicity of infection (MOI) of 10 and the efficiency of transfection was detected by FACS. Neurospheres dedifferentiated from Müller cells were divided into three groups: Group A: neurospheres transfected by PGC-FU-Atoh7-GFP; Group B: neurospheres transfected by empty vector PGC-FU-GFP; and Group C: neurospheres without transfection. After transfection, the cells were plated onto 0.01% poly-D-lysine (Sigma)-coated 24-mm coverslips (Corning) at a concentration of 1 × 10^4^ cells/well, and cultured in 1 ml differentiation medium supplemented with brain-derived neurotrophic factor (BDNF: brain-derived neurotrophic factor) (1 ng/ml) (Peprotech), RA (1 μM) (Sigma) and 1% FBS at 37°C in a 5% CO_2_ incubator, and the medium was changed 48 h after plating to remove debris. Thereafter, the cells were fed every two days by replacing one third of the differentiation medium. At days 7 and 14, the cells were fixed by cold 4% paraformaldehyde for immunocytochemical analysis to calculate the percentage of ganglion cells.

### Interference of Brn-3bsiRNA, Isl-1siRNA and Notch signal pathway inhibitor

The purified neurospheres were collected and dissociated with Accutase: Sigma, St. Louis, MO, USA, and the stem cells were seeded into six-well culture plates at a density of 1 × 10^5^ cells/well, and cultured in 2 ml differentiation medium supplemented with BDNF (1 ng/ml), RA: retinoic acid (1 μM) and 1% FBS. The cells were randomly divided into groups as follows: Group a1: Brn-3b siRNA group (sc-38767), Group a2: Control siRNA group (scrambled sequence, sc-37007), Group a3: Control group (without any handling); Group b1: Isl-1 siRNA group (sc-37122), Group b2: Control siRNA group (scrambled sequence, sc-44230), Group b3: Control group (without any handling); Group c1: Notch signal pathway inhibitor (GSI) group (sc-221656), Group c2: Control group (without any handling). The stem cells were transfected with the indicated siRNA or treated with GSI. After 72 h, the cells were transfected with lentivirus PGC-FU- Atoh7-GFP. At seven days after transfection, immunofluorescence staining was performed to detect the percentage of ganglion cells in total differentiated cells.

### RT-PCR analysis

Total RNA was isolated from cells using Trizol: Sigma, St. Louis, MO, USA reagent according to the manufacturer’s protocol, and reverse transcribed to cDNA. PCR reaction was performed in a 20 μl volume containing the following: 10 μl 2 × SYBR Green mix, 1 μl 10 μM forward primer and reverse primer (the primers were listed in Table 2), 2 μl diluted cDNA and 6 μl double-distilled H_2_O. Amplification conditions were as follows: 15 sec at 95°C (one cycle); 5 sec at 95°C, 5 sec at annealing temperature and 30 sec at 72°C (45 cycles). The results were analyzed by ABIViia7: ABI, Foster City, CA, USA.

**Table 2:** List of primers used in this study

**Name**	**Sequence**	**Annealing temp (°C)**	**Size (bp)**	**Acc. no**
Glutamine synthetase	Forward: 5′TCACAGGGACAAATGCCGAG3′	58	362	M96152
	Reverse: 5′GTTGATGTTGGAGGTTTCGTGG3′			
Vimentin	Forward: 5′AAGGCACTAATGAGTCCCTGGAG3′	56	251	NM031140
	Reverse: 5′GTTTGGAAGAGGCAGAGAAATCC3′			
CRALBP	Forward: 5′CTGAGTTTGGAGGAATCTTGC3′	54	150	XM217702
	Reverse: 5′TGGATTTGGGGGAGAGTTC3′			
Clusterin	Forward: 5′CCTCCAGTCCAAGATGCTCAAC3′	58	292	NM_053021
	Reverse: 5′TTTCCTGCGGTATTCCTGTAGC3′			
Carbonic anhydrase	Forward: 5′TTGCCAATGGAGACCGACAG3′	58	233	NM_019291
	Reverse: 5′TGAGCCCCAGTGAAAGTGAAAC3′			
Opsin	Forward: 5′CATGCAGTGTTCATGTGGGA 3′	64	422	U22180
	Reverse: 5′AGCAGAGGCTGGTGAGCATG 3′			
mGluR6	Forward: 5′CACAGCGTGATTGACTACGAG3′	56	317	D13963
	Reverse: 5′CTCAGGCTCAGTGACACAGTTAG3′			
HPC1	Forward: 5′AAGAGCATCGAGCAGCAGAGCATC3′	60	342	NM016801
	Reverse: 5′CATGGCCATGTCCATGAACAT3′			
Brn-3b	Forward: 5′GGCTGGAGGAAGCAGAGAAATC 3′	60	141	AF390076
	Reverse: 5′TTGGCTGGATGGCGAAGTAG 3′			
CD31	Forward: 5′AAGAGCAACTTCCAGACCGTCC 3′	58	222	NM_031591
	Reverse: 5′AAGCACCATTTCATCTCCAGACTG 3′			
Tyrosinase	Forward: 5′TCAGTCTATGTCATCCCCACAGG3′	56	252	NM_011661
	Reverse: 5′GTTCTCATCCCCAGTTAGTTCTCG3′			
Nestin	Forward: 5′TGGAGCAGGAGAAGCAAGGTCTAC3′	56	295	NM012987
	Reverse: 5′TCAAGGGTATTAGGCAAGGGGG3′			
Pax6	Forward: 5′CCATCTTTGCTTGGGAAATCC3′	56	310	NM_013001
	Reverse: 5′TCATCCGAGTCTTCTCCATTGG3′			
Atoh7	Forward: 5′ATGAAGTCGGCCTGCAAAC3′	55	389	AF_071223
	Reverse: 5′GGGTCTACCTGGAGCCTAGC3′			
Notch1	Forward: 5′TCTGGACAAGATTGATGGCTACG3′	56	329	NM008714
	Reverse: 5′CGTTGACACAAGGGTTGGACTC3′			
β-Actin	Forward: 5′GTGGGGCGCCCCAGGCACCA 3′	50	548	XM_037235
	Reverse: 5′ CTCCTTAATGTCACGCACGATTTC 3′			

### Western blot analysis

Proteins were extracted from the cells by using Radio Immuno Precipitation Assay (RIPA: RiboBio: Guangzhou, Guangdong, China) buffer containing 1:100 protease inhibitor and 1:100 phosphatase inhibitor. The protein concentration was determined by using a microplate reader. Lysates were separated on SDS-PAGE and transferred onto polyvinylidene fluoride (PVDF) membranes. The membranes were blocked with 5% nonfat milk in TBS plus 0.1% Tween (TBS-T) for 1 h, then incubated with primary antibodies for 1 h at room temperature. After several washes, the membranes were incubated with HRP-conjugated secondary antibodies for 1 h. Blots were visualized with ECL: Enhanced Chemiluminescence buffer.

### Statistical analysis

All data were expressed as the mean ± SD. Statistical analysis was performed with one-way ANOVA and Student’s *t*- test using SPSS: Chicago, IL, USA 13.0. *P* <0.05 was considered significant.

## Results

### Purification and morphological characteristics of Müller cells

**Figure 1:**
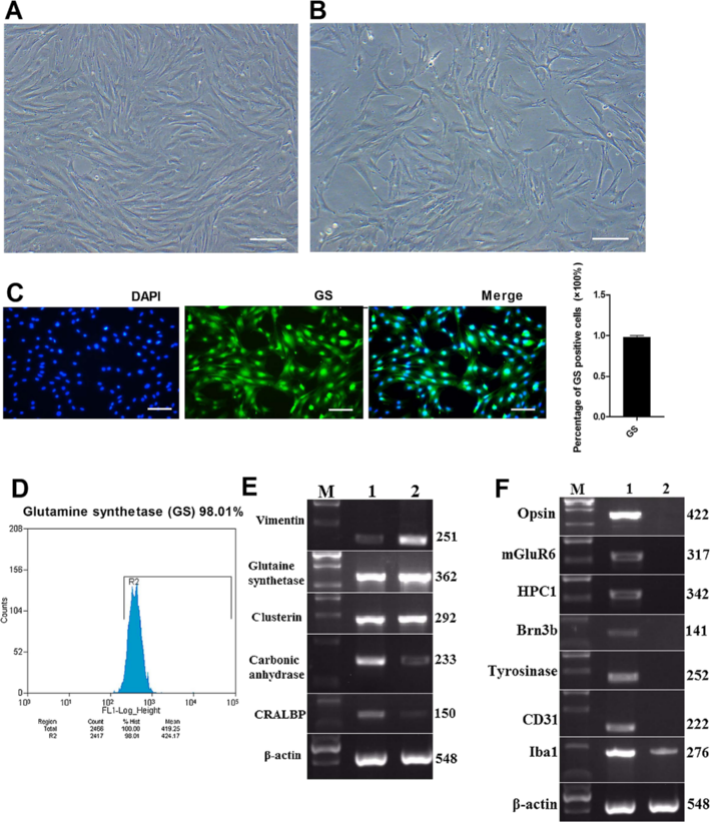
**Purity of enriched Müller cells.** Rat retinal Müller cells were enriched and passaged to obtain a highly purified cell population **(A, B)**. Scale bar: 100 μm. Immunocytochemical and FACS analysis showed that more than 98% of purified cells were immunoreactive to Müller cell marker GS **(C, D)**. Scale bar: 100 μm. The purity of enriched cells was evaluated by RT-PCR analysis to detect the expression of Müller cell specific transcripts **(E)** and other cell type specific transcripts **(F)**. Lane M: DNA marker; lane 1: PN21 retina; lane 2: purified Müller cells. FACS, fluorescence-activated cell sorting.

After the dissociated and trypsinized retinal tissues were filtrated by a 200-mesh filter, retinal cells were inoculated in a 25 cm^2^ culture flask. About 72 h later, we found that some of retinal cells attached to the bottom of the flask. The cells initially appeared to be cell aggregates, single cells or debris. After five to seven days of culture, numerous bipolar spindle-shaped cells were observed on the surface of the flask. At 8 to 10 days, the cell culture medium became light yellow and the culture medium was replaced every 2 to 3 days; both aggregated and loosely attached materials were discarded, leaving only tightly adherent cells on the flask surface. As the adherent cells proliferated, they became increasingly flattened and epithelioid shaped. When the proliferated cells formed a complete confluent monolayer of epithelioid cells (Figure 1A), the cells were trypsinized and subcultured in DMEM containing 20% FBS for another five to six days to obtain a further purified Müller cell population. After the fourth passage, these cells were the same size and shape, and had abundant cytoplasm and well-defined membranes (Figure 1B). To determine whether the cultured cells were Müller cells, we chose the cells in the fourth passage to examine immunoreactivity for glutamine synthetase (GS) (Müller cell specific markers) [21]. The results showed that 98.10 ± 2.18% of the cells in the monolayer culture were immunoreactive for GS (Figure 1C). To further determine the purity of Müller cell culture, we carried out FACS and RT-PCR analysis. FACS showed that 98.01% of purified cells were immunoreactive for GS (Figure 1D). RT-PCR analysis revealed that cells in the culture expressed a battery of transcripts characteristic of Müller cells, such as GS, vimentin, cellular retinaldehyde-binding protein (CRALBP), clusterin and carbonic anhydrase [9,22] (Figure 1E). In contrast, the transcripts corresponding to rod photoreceptors (opsin), bipolar cells (mGluR6), amacrine cells (Syntaxin 1), retinal ganglion cells or RCGs (Brn3b), endothelial cells (CD31) and retinal pigmented epithelium (RPE)/pigmented ciliary epithelium (tyrosinase) were not detected, suggesting that the monolayer culture was enriched with Müller cells and not contaminated with the above mentioned cells (Figure 1F). However, low levels of transcripts characteristic of microglia (Iba1) were detected, suggesting the presence of microglia in the enriched Müller cell culture.

### Dedifferentiation of purified Müller cells into retinal stem cells

**Figure 2:**
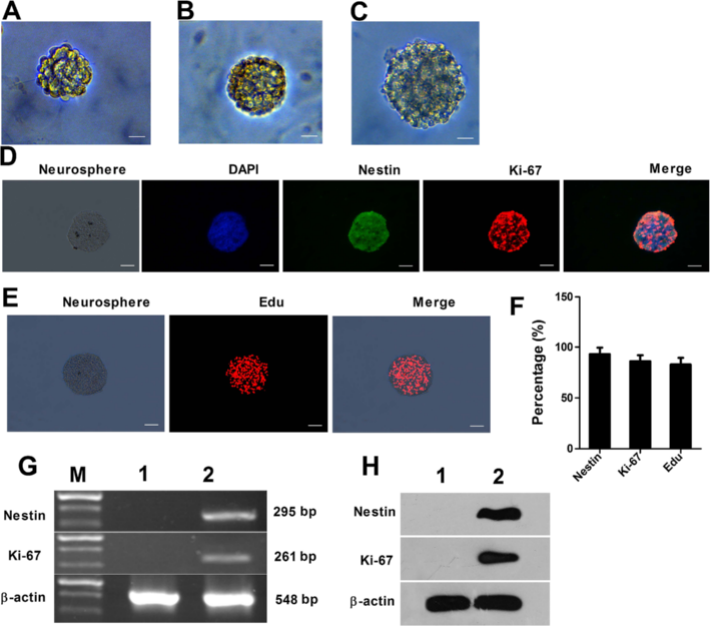
**Stem cell properties of enriched Müller cells.** Enriched Müller cells exposed to the stem cell-conditioned medium formed neurospheres **(A, B)**. The neurospheres were passaged to get new clonal neurospheres **(C)**. Immunofluorescence staining showed that the stem cells within the cell spheres had positive expression of retinal stem cell-specific markers Nestin (92.94 ± 6.48%) and Pax6 (85.96 ± 6.04%) **(D, F)**. Immunocytochemical analysis of Edu showed that newborn cell spheres had the capacity of effective proliferation (82.80 ± 6.65%) **(E, F)**. Scale bar: 100 μm. RT-PCR and Western blot analysis to detect the expression of stem cell markers Nestin and Pax6 **(G, H)**. Lane M: DNA marker; lane 1: Müller cells; lane 2: neurospheres.

The purified Müller cells were cultured in stem cell-conditioned medium for three days, some cells underwent apoptosis, some cell processes became smaller and the cell body became round. The proliferation was clonal, and a few spherical or mulberry-shaped cell spheres composed of dozens of cells appeared (Figure 2A). At five to seven days of culture, the cell spheres increased in both number and size, cells displayed good refraction and exhibited well-defined cell boundaries at the edge of cell spheres and the cell spheres became further rounded, resembling neurospheres (Figure 2B). Thereafter, the cell spheres showed no significant increase in number and size. After seven days, the neurospheres were collected and dissociated into single stem cells which were cultured in serum-free dedifferentiation media to generate new clonal neurospheres (Figure 2C). Immunofluorescence staining showed that stem cells within the spheres were positive for retinal stem cell-specific markers Nestin (green fluorescence, 92.94 ± 6.48%) and Pax6 (red fluorescence, 85.96 ± 6.04%) (Figure 2D, F), suggesting that retinal Müller cells can dedifferentiate into retinal stem cells in the conditioned medium. Meanwhile, Edu staining showed that most of the nuclei within the cell spheres were stained red (82.80 ± 6.65%), suggesting that the new cell spheres have the capacity for effective proliferation (Figure 2E, F). RT-PCR analysis showed that cell spheres in the culture expressed a battery of transcripts characteristic of stem cells, such as Nestin and Pax6, which were absent in the Müller cells (Figure 2G). Western blot analysis further confirmed the expression of Nestin and Pax6 in the cell spheres but not in the Müller cells (Figure 2H). Taken together, these data suggest that Müller cells were dedifferentiated into retinal stem cells. 

### Atoh7 promotes the differentiation of Müller cells-derived stem cells into ganglion cells

**Figure 3:**
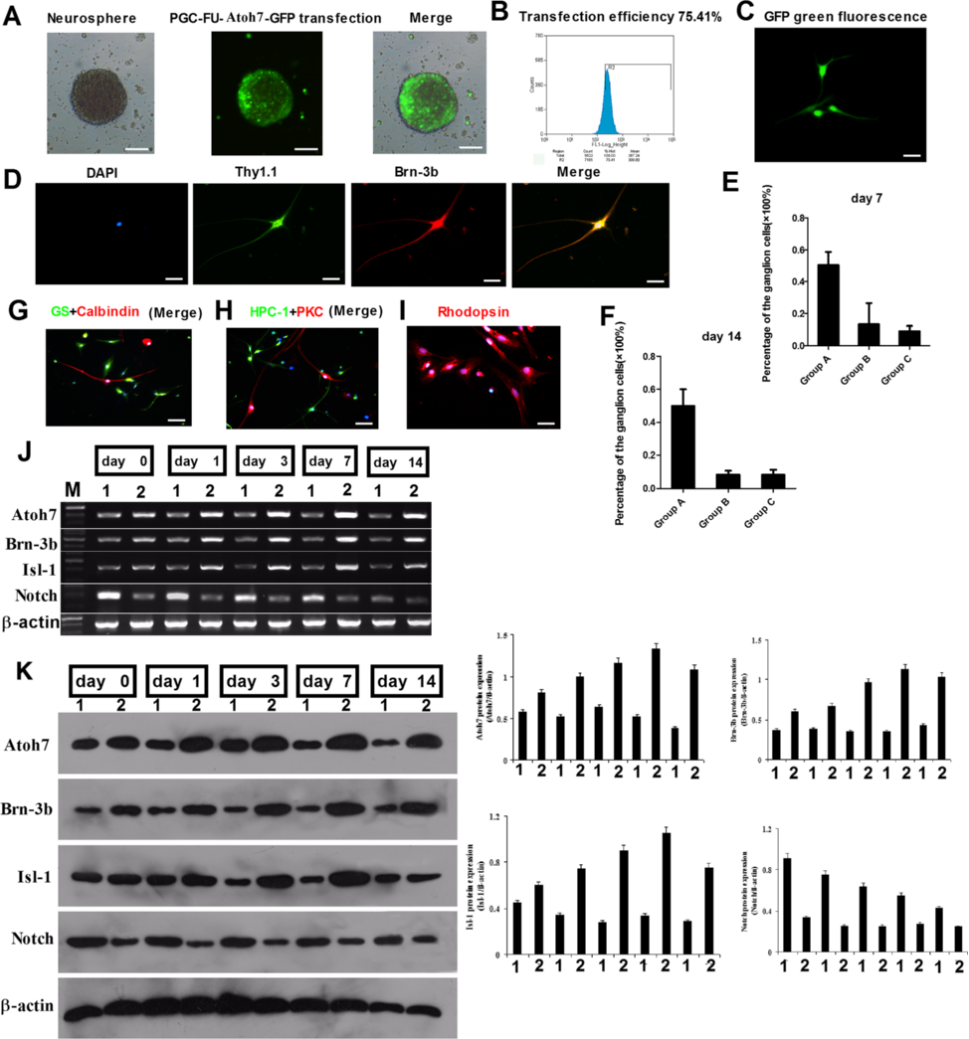
**Dedifferentiation of retinal stem cells.** The stem cells differentiated from retinal Müller cells were transfected with lentivirus PGC-FU-Atoh7-GFP **(A)**. FACS analysis showed that the transfection efficiency was 75.41% at 48 h after transfection **(B)**. The GFP green fluorescence could be seen perspicuously in differentiated cells **(C)**. Double immunocytochemical analysis showed that ganglion cells expressed both Thy1.1 and Brn-3b **(D)**. Scale bar: 100 μm. Ten different visual fields (×100) in each group were selected to count the percentage of ganglion cells. The percentage of ganglion cells was similar between Group B and Group C (13.3 ± 13.25% versus 9.10 ± 3.21%), but significantly higher in Group A (50.40 ± 8.22%) **(E)**. At 7 and 14 days, the percentage of ganglion cells was similar **(E, F)**. Apart from ganglion cells, retinal stem cells differentiated into other type of cells such as amacrine cells, horizontal cells, bipolar cells, photoreceptor cells and Müller cells **(G-I)**. Scale bar: 100 μm. RT-PCR and Western blot analysis showed that the expression of Atoh7, Brn-3b and Isl-1 was increased after lentivirus PGC-FU-Atoh7-GFP transfection, but Notch1 expression was slightly decreased **(J, K)**. Lane M: DNA marker; lane 1: groups without transfection; lane 2: groups with transfection by lentivirus PGC-FU-Atoh7-GFP. FACS, fluorescence-activated cell sorting.

Next, stem cells dedifferentiated from Müller cells were transfected with lentivirus PGC-FU- Atoh7-GFP or empty vector PGC-FU-GFP. After 48 h, the number of GFP labeled cells increased and fluorescence intensity was enhanced and distributed homogeneously in the cytoplasm (Figure 3A). FACS analysis showed that the transfection efficiency was 75.41% (Figure 3B). At this time, the neurosphere cells of the three groups were separated into single cells by Accutase and green fluorescence could be seen perspicuously in the single cells using fluorescent microscopy (Figure 3C).

After culture of the single cells in differentiation medium for seven days, immunocytochemical analysis was performed to calculate the percentage of the ganglion cells, which were positive for Thy1.1 (green fluorescence) and Brn-3b (red fluorescence) (Figure 3D). The percentage of ganglion cells was similar between cells transfected with PGC-FU-GFP and cells untransfected (13.3 ± 13.25% versus 9.10 ± 3.21%), significantly lower than that in cells transfected with PGC-FU-Atoh7-GFP (50.40 ± 8.22%) (Figure 3E). Collectively, our results indicate that Atoh7 promotes the differentiation of Müller cells-derived stem cells into ganglion cells.

At 14 days after transfection, the cells were examined again for ganglion cell-specific markers. Immunocytochemical analysis showed that the percentage of ganglion cells was 8.40 ± 2.32% (Group B), 8.20 ± 3.19% (Group C) and 49.90 ± 10.16% (Group A) (Figure 3F). These results showed that at 7 and 14 days, the efficiency of Atoh7 to promote the differentiation of retinal stem cells into retinal ganglion cells was similar (50.40 ± 8.22% versus 49.90 ± 10.16%). Apart from ganglion cells, retinal stem cells derived from Müller cells were differentiated into other types of cells, such as amacrine cells, horizontal cells, bipolar cells, photoreceptor cells and Müller cells (Figure 3G-I).

RT-PCR and Western blot analysis showed that the expression of Atoh7, Brn-3b and Isl-1 at both mRNA and protein levels were increased on 0 day, 1 day, 3 days, 7 days and 14 days after transfection with PGC-FU-Atoh7-GFP, but the expression of Notch1 was slightly reduced. The control group (the cell spheres with no transfection) showed no significant differences in mRNA and protein expression of Atoh7, Brn-3b, Isl-1 and Notch1 (Figure 3J, K). These data suggest that Atoh7 regulates the expression of Brn-3b, Isl-1 and Notch1 in retinal stem cells.

### Effects of Brn-3bsiRNA, Isl-1siRNA and Notch inhibitor on the differentiation of Müller cells-derived stem cells into ganglion cells

**Figure 4:**
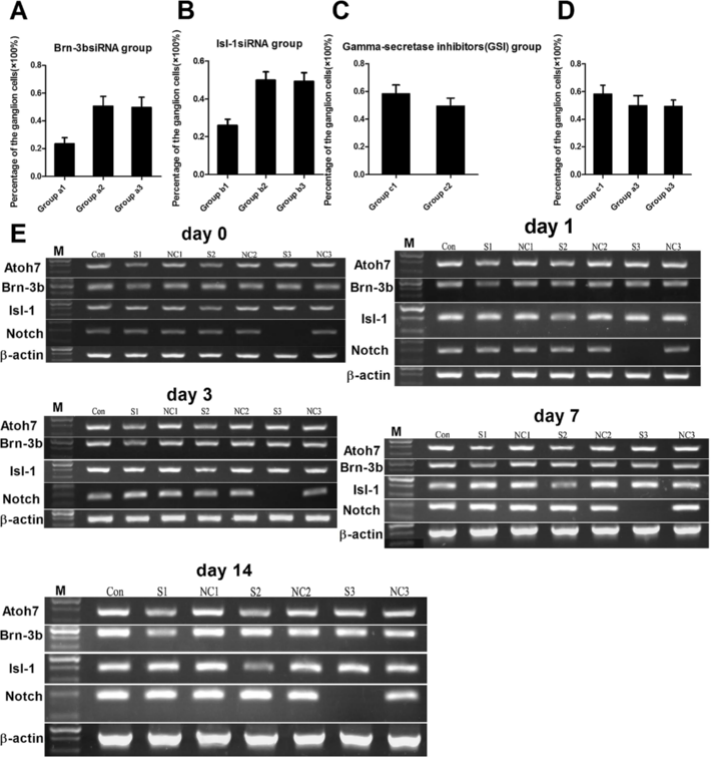
**The percentage of ganglion cells differentiated from retinal stem cells. A**. The percentage of ganglion cells differentiated from retinal stem cells was 23.3 ± 4.45% (group a1), 50.6 ± 7.04% (Group a2) and 49.7 ± 7.36% (Group a3). **B**. The percentage of ganglion cells differentiated from retinal stem cells was 25.9 ± 3.35% (Group b1), 49.9 ± 5.38% (Group b2) and 49.2 ± 4.64% (Group b3). **C**. The percentage of ganglion cells differentiated from retinal stem cells was 58.2 ± 6.46% (Group c1) and 49.4 ± 5.78% (Group c2). **D**. Atoh7 and gamma-secretase inhibitor (GSI) were synergistic to promote the differentiation of retinal stem cells derived from Müller cells into retinal ganglion cells (F = 6.533, *P* = 0.005). **E**. RT-PCR showed that the mRNA expression of Brn-3b, Isl-1 and Notch1 was reduced in Brn-3b siRNA group, Isl-1 siRNA group and GSI group, respectively, at different time points (0 day, 1 day, 3 days, 7 days, 14 days). Atoh7 and GSI synergistically inhibited mRNA expression of Notch1.

To explore the signaling mechanisms by which Atoh7 promotes the differentiation of Müller cells-derived stem cells into ganglion cells, we treated retinal stem cells with Brn-3b siRNA, Isl-1 siRNA or gamma-secretase inhibitors (GSI). After 72 h, the stem cells were transfected by PGC-FU-Atoh7-GFP. After seven days, the cells in each group were evaluated for ganglion cell-specific markers. The percentages of ganglion cells were as follows: 23.30 ± 4.45% (Group a1), 50.60 ± 7.04% (Group a2) and 49.70 ± 7.36% (Group a3) (Figure 4A); 25.90 ± 3.35% (Group b1), 49.9 ± 5.38% (Group b2) and 49.2 ± 4.64% (Group b3) (Figure 4B); 58.2 ± 6.46% (Group c1) and 49.4 ± 5.78% (Group c2) (Figure 4C). These results showed that knockdown of Brn-3b or Isl-1 could inhibit the differentiation of Müller cells-derived stem cells into retinal ganglion cells, while the Notch signal pathway inhibitor was able to promote the differentiation into retinal ganglion cells. In addition, Atoh7 and GSI exhibited synergistic effects to promote the differentiation of Müller cells-derived stem cells into retinal ganglion cells (between Group c1 and Group a3, b3; F = 6.533, *P* = 0.005; Figure 4D).

**Figure 5:**
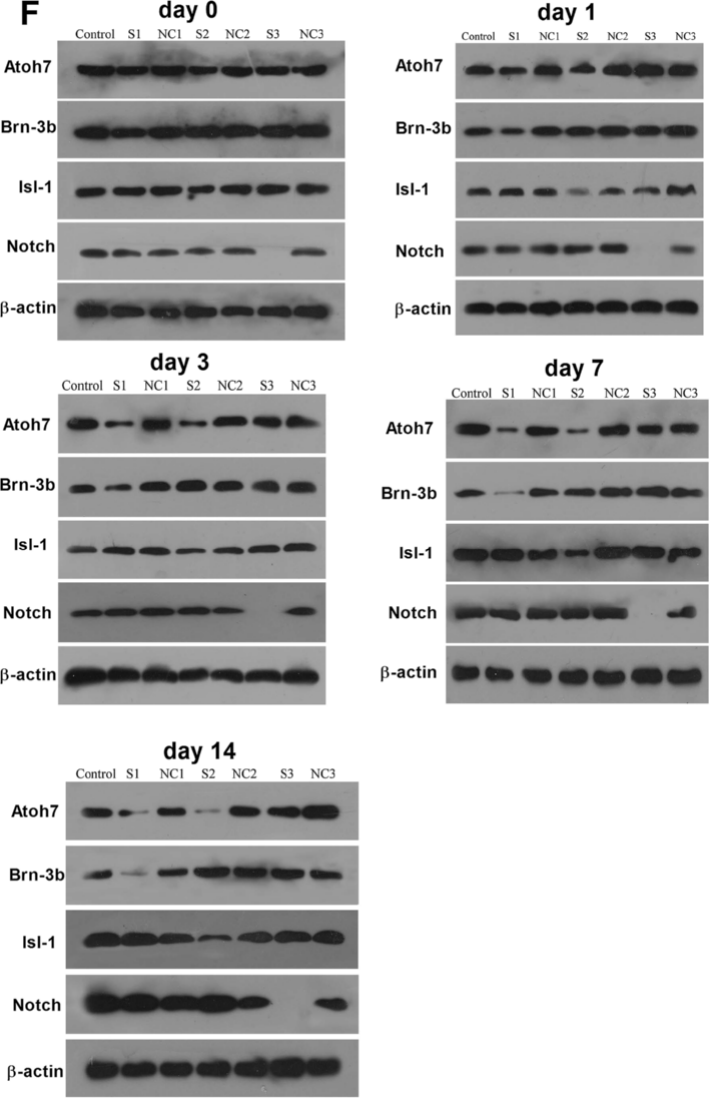
**Atoh7 and GSI synergistically inhibit protein expression of Notch1 in retinal stem cells.** The protein expression of Brn-3b, Isl-1 and Notch1 was reduced in Brn-3b siRNA group, Isl-1 siRNA group and GSI group, respectively, at different time points (0 day, 1 day, 3 days, 7 days, 14 days). Lane M: DNA marker; lane Con = retinal stem cells untreated; lane S1: retinal stem cells transfected with Brn-3b siRNA and PGC-FU-Atoh7-GFP; lane NC1: retinal stem cells transfected with scrambled siRNA and PGC-FU-Atoh7-GFP; lane S2: retinal stem cells transfected with Isl-1 siRNA and PGC-FU-Atoh7-GFP; lane NC2: retinal stem cells transfected with scramble siRNA and PGC-FU-Atoh7-GFP; lane S3: retinal stem cells treated with GSI and transfected with PGC-FU-Atoh7-GFP; lane NC3: retinal stem cells transfected with PGC-FU-Atoh7-GFP. β–actin was loading control.

RT-PCR and Western blot analysis showed that the expression of Brn-3b, Isl-1 and Notch1 was reduced in the Brn-3b siRNA group, the Isl-1 siRNA group and the GSI group, respectively. In addition, Atoh7 and GSI synergistically inhibited the expression of Notch1 (Figures 4E and 5).

## Discussion

Glaucoma is a complex, multivariate, irreversible blinding eye disease. The blockade of retinal ganglion cell apoptosis, the reduction of intraocular pressure, and nourishment of the optic nerve are known to be somewhat effective in prolonging the life of ganglion cells and retarding disease progression for patients with early glaucoma or progressing glaucoma. However, such treatment strategies are ineffective for patients with advanced glaucoma. This discrepancy lies in the fact that most or all retinal ganglion cells have undergone apoptosis in patients with advanced glaucoma, and the number of surviving ganglion cells is too few to reverse pathological changes resulting from glaucoma. Therefore, there is an urgent need to develop novel strategies to regenerate retinal ganglion cells to reverse disease progression or even restore the vision.

Stem cell engineering has emerged as a promising approach for retinal regeneration therapy [23,24]. By incorporating stem cells into the retina and inducing their proliferation and differentiation into target cells, it is possible to replenish retinal neurons and restore retinal function. Retinal Müller cells, the glial cells in the retina, retain proliferation potential and offer an abundant source for cell engineering [9,10]. In addition, Müller cells span the entire width of the retina and are widely distributed among ganglion cells. This increases the chance to better integrate the cells converted from Müller cells into the ganglion cell layer. All these features suggest that retinal Müller cells are the most promising source of stem cells in the are features suggest that retinal Müller cells are the most promising source of stem cells in the treatment of glaucoma. Although there is substantial evidence that retinal Müller cells can dedifferentiate into retinal stem cells in certain conditions, the potential of their differentiation into ganglion cells remains unclear. Therefore, in the present study, we selected stem cells dedifferentiated from rat retinal Müller cells as the target cells. We successfully transfected these stem cells with lentivirus pGC-FU-Atoh7-GFP, and demonstrated that Atoh7 could promote the differentiation of Müller cells derived stem cells into ganglion cells.

Glutamine synthetase (GS) is a key enzyme that transfers glutamic acid into glutamine and is only expressed in Müller cells [21]. Therefore, we chose GS as the specific marker to identify Müller cells. The results showed that the cells isolated from rat retina displayed the general morphology of Müller cells and more than 98.10 ± 2.18% of the cells were immunopositive for GS. FACS showed that 98.01% of the purified cells were immunoreactive for GS. RT-PCR revealed that the cells expressed a battery of transcripts characteristic of Müller cells, such as GS, vimentin, CRALBP, clusterin and carbonic anhydrase. In contrast, transcripts corresponding to rod photoreceptors, bipolar cells, amacrine cells, retinal ganglion cells, endothelial cells and RPE/pigmented ciliary epithelium were not detected. Taken together, these results demonstrate that our method of purifying Müller cells is effective.

Currently, two methods are commonly used to induce the dedifferentiation of retinal Müller cells: one is to use ouabain, N-methyl-D-aspartate (NMDA) or other agents to induce the effects of injury, and the other is to add various cell growth factors [11-13]. In the present study, we chose a serum-free DMEM/F12 medium supplemented with EGF, bFGF and other cytokines to induce the dedifferentiation of retinal Müller cells. Characterization of the cell spheres dedifferentiated from retinal Müller cells showed that the cells within the neurospheres highly expressed retinal stem cell-specific markers Nestin and Pax6, which, however, are not expressed in normal Müller cells. Furthermore, positive Edu staining, an important indicator of proliferation, was observed in 82.80 ± 6.65% of the cells, proving that the cells derived from dedifferentiation have the proliferative ability of stem cells. These data suggest that upon cytokine stimulation, Müller cells acquire the property of neural stem cells.

The serum is a natural inducer of stem cell differentiation, but previous study showed that stem cells dedifferentiated from retinal Müller cells did not undergo neuronal cell differentiation when cultured in medium only containing serum [25]. BDNF and RA have been shown to induce neural stem cells to differentiate into neurons and promote their maturation [26,27]. Thus, we chose to induce re-differentiation of stem cells by using a culture medium supplemented with BDNF and RA. We found that the stem cells had the ability to re-differentiate. Cells of various shapes were obtained as early as at days 7 to 10 of culture. Even without transfection with Atoh7 expression vector, ganglion cells induced by our medium could account for 9.10 ± 3.21% of the total differentiated cells.

The directed differentiation of retinal stem cells is mainly co-regulated by the extracellular microenvironment factors and endogenous cytokines. The bHLH (basic helix-loop-helix) family plays an important role in regulating retinal cell differentiation [28]. Atoh7 is a member of the bHLH family and its expression pattern is consistent with the spatiotemporal pattern of retinal ganglion cell differentiation [28]. Atoh7 is a key regulatory factor essential for the development of retinal ganglion cells in vertebrates [16]. Ectopic expression of Atoh7 has been shown to increase the number of retinal ganglion cells differentiated from stem cells [17]. Therefore, in the present study, we transfected Müller cells-derived stem cells with the Atoh7 expression vector to promote the differentiation into ganglion cells. The results showed that transfection with lentivirus PGC-FU-Atoh7-GFP led to the differentiation into ganglion cells at a frequency nearly five times that of transfection with empty vector, confirming the crucial role of Atoh7 in promoting the directed differentiation of stem cells into ganglion cells.

To explore the signaling mechanisms by which Atoh7 promotes the differentiation of Müller cells-derived stem cells into ganglion cells, we employed the loss of function approach to intervene with the expression of Brn-3b, Isl-1 and Notch1. Our results showed that knockdown of Brn-3b or Isl-1 inhibited the differentiation of Müller cells-derived stem cells into retinal ganglion cells, while Notch signal pathway inhibitor GSI promoted the differentiation into retinal ganglion cells. These results suggest that Brn-3b and Isl-1 promote while Notch signaling inhibits the differentiation of Müller cells-derived stem cells into retinal ganglion cells. In particular, we found that overexpression of Atoh7 and inhibition of Notch signaling by GSI exhibited synergistic effects to promote the differentiation into retinal ganglion cells. Considering our results that Atoh7 inhibited the expression of Notch1, we propose that Atoh7 promotes the differentiation of retinal stem cells derived from Müller cells into retinal ganglion cells by inhibiting Notch signaling.

## Conclusions

We established an effective method to isolate and purify Müller cells from rat retina and successfully induced them to dedifferentiate into retinal stem cells. Ectopic expression of Atoh7 promoted the expression of Brn-3b and Isl-1 but inhibited the expression of Notch1 in these cells. In addition, knockdown of Brn-3b or Isl-1 inhibited, while GSI promoted the differentiation of these stem cells into retinal ganglion cells. These data suggest that Atoh7 promotes the differentiation of Müller cells-derived retinal stem cells into retinal ganglion cells by inhibiting Notch signaling, and open up a new avenue for gene therapy and optic nerve regeneration in glaucoma.

## Abbreviations

BDNF: Brain-derived neurotrophic factor; bHLH: Basic helix-loop-helix; BSA: Bovine serum albumin; CRALBP: Cellular retinaldehyde-binding protein; FACS: Fluorescence-activated cell sorting; FBS: Fetal bovine serum; DMEM: Dulbecco’s modified Eagle’s medium; GS: Glutamine synthetase; GSI: Gamma-secretase inhibitor; IOP: Intraocular pressure; MOI: Multiplicity of infection; NMDA: N-methyl-D-aspartate; PBS: Phosphate buffer solution; PVDF: Polyvinylidene fluoride; RGCs: Retinal ganglion cells; RIPA: Radio Immuno Precipitation Assay; SD rats: Sprague Dawley rats; SD: Standard deviation; RPE: Retinal pigment epithelium; TBS-T: Tris buffered saline plus 0.1% Tween.

## Competing interests

The authors declare that they have no competing interests.

## Authors’ contributions

WS and XZ carried out the experiments. WS drafted the manuscript. XX conceived of the study, and participated in its design and coordination, and helped to draft the manuscript. All authors read and approved the final manuscript.
